# Cyclophilin A is Associated with Peripheral Artery Disease and Chronic Kidney Disease in Geriatrics: The Tianliao Old People (TOP) Study

**DOI:** 10.1038/srep09937

**Published:** 2015-04-24

**Authors:** Meng-Chuan Liu, Yen-Wei Lee, Po-Tseng Lee, Chin-Sung Chang, Yun-Lin Tai, Jia-Rong Yu, Xiao-Ting Su, Ling-Wei Hsu, Sheng-Hsiang Lin, Chi-Hsing Wu, Ping-Yen Liu

**Affiliations:** 1 School of Medicine, College of Medicine, National Cheng Kung University, Tainan, Taiwan; 2 Division of Cardiology, Department of Internal Medicine, National Cheng Kung University Hospital, Tainan, Taiwan; 3 Institute of Clinical Medicine, National Cheng Kung University, Tainan, Taiwan; 4 Department of Family Medicine, National Cheng Kung University Hospital, Tainan, Taiwan; 5 Institute of Basic Medical Sciences, National Cheng Kung University, Tainan, Taiwan

## Abstract

Cyclophilin A (CyPA), secreted by vascular smooth muscle cells in response to oxidative stress, is important in the pathogenesis of progressive peripheral arterial occlusion disease (PAOD), which is common among chronic kidney disease. We explored the prevalence of PAOD in Taiwan’s elderly (≥ 65 years old) population and its association with CyPA and renal function. Residents of Tianliao District, a rural community in southern Taiwan, were surveyed. An ankle-brachial index (ABI) < 0.91 was defined as PAOD. Chronic kidney disease (CKD) was defined based on eGFR levels < 60 mL/min/1.73 m^2^. Serum CyPA was measured. Of the 473 participants, 68 (14.4%) had PAOD. Multiple logistic regression analysis showed PAOD was significantly associated with lower eGFR, lower BMI, higher glycated hemoglobin and higher pulse pressure. Serum CyPA levels in participants with PAOD were significantly higher than those with normal ABI values (47.3 ± 0.4 vs. 25.5 ± 0.2 ng/mL, p < 0.001). Moreover, eGFR inversely correlated with serum CyPA level (p < 0.05) in participants with CKD, but not in participants with normal renal function. In conclusion, with a prevalence of PAOD as high as 14.4% in an elderly community, CyPA might be the link between PAOD and advanced impaired renal function.

Peripheral arterial occlusive disease (PAOD) is an atherosclerotic disease with lesions in the lower extremities in the majority, and the classic manifestation is intermittent claudication. Patients with PAOD not only have reduction in quality of life, but also have a substantial risk of cardiovascular morbidity and death^1^. The prevalence of PAOD increases markedly with older age^2^, which is a threat to an aging community.

Even though intermittent claudication is a classic symptom of PAOD, it was absent in most of our patients. However, they manifested severe atherosclerotic changes when they were finally diagnosed^3^. The ankle-brachial index (ABI), which is defined as a ratio with systolic pressure in the ankle divided by the systolic pressure in the arm, has been recognized as a useful diagnostic tool for early PAOD detection, with or without symptoms^4^.

Traditional atherosclerotic risk factors—smoking, diabetes mellitus (DM), hyperlipidemia, and hypertension (HTN)—are associated with a higher prevalence of PAOD^3^. CKD is an important disease in Taiwan, which has the highest incidence of end-stage renal disease (ESRD) in the world^5^. Whether CKD is an independent or covariant risk disease for PAOD is unclear. The 1987–89 Atherosclerosis Risk in Communities (ARIC) study^6^ reported that CKD was associated with a higher incidence of PAOD, and two other studies^7^,^8^ reported that CKD was associated with both a high and a low ankle-brachial index (ABI), and that patients with CKD and a low ABI had a more rapid decline in renal function^8^.

CyPA is a cyclophilin that belongs to a family of immunophilins. CyPA binds to cyclosporine A^9^,^10^ and is an immunosuppressant that protects against rejection after internal organ transplants. In addition to being an intracellular multifunctional chaperone in organs, after it is secreted from vascular smooth muscle cells (VSMCs) under oxidative stress, CyPA also has extracellular functions^11^,^12^. It can, for example, promote the development of atherosclerosis in several ways: by facilitating the proliferation and migration of VSMCs, activating proinflammatory pathways in endothelial cells, being a chemoattractant, and augmenting the production of ROS^13^,^14^,^15^,^16^.

Patients with significant coronary stenosis have higher plasma CyPA levels; thus, CyPA has become a biomarker for coronary artery disease^17^. It is also associated with advanced CKD. How CyPA functions in the pathogenesis of PAOD is unclear. Therefore, we examined whether patients with PAOD have a higher serum CyPA level, which we hypothesized was the link between PAOD and traditional and nontraditional cardiovascular risk factors, including CKD.

## Methods

### Study population and data collection

This community-based study was conducted in July 2012, after the Tianliao Old People (TOP) study^18^,^19^,^20^,^21^. Tianliao District is a rural and aging community in southern Taiwan’s Kaohsiung City. According to the census^18^,^19^,^20^,^21^, the number of elderly (≥ 65 years old) residents in 2012 was 1966 (25.2%), a significantly higher proportion than in Taiwan’s general population.

We surveyed 549 elderly voluntary participants in this community. Almost half (n = 868, 44.15%) of the elderly were excluded for a variety of reasons: registered addresses for 489 residents were empty houses, 40 residents had died, 138 were not ambulatory, and 201 were otherwise unreachable. Finally, 549 (response rate: 50%) residents agreed to participate; each signed an informed consent before the study began. The protocol was approved by the Institutional Review Board of our hospital (IRB number: B-ER-101-119) and all participants signed the inform consent before they were enrolled in this study. Besides, the methods were carried out in accordance with the approved current guidelines. Participants were significantly younger than non-participants (76.0 ± 6.2 years *vs.* 76.8 ± 7.4 years, p = 0.001), but there were no other significant background differences between them (data not shown).

Each participant was interviewed by well-trained interviewers based on a structural questionnaire and was asked about their basic characteristics, habits, and medical history. Blood samples were collected from the participants after they had fasted for at least 8 hours. Cyclophilin A (CyPA), hemoglobin A1c (HbA1c), high-sensitivity C-reactive protein (hsCRP), serum creatinine concentration, total cholesterol, high-density lipoprotein-cholesterol (HDL-C), low-density lipoprotein-cholesterol (LDL-C), triglyceride levels, ABI, body weight, and standing height were measured and recorded.

After we excluded 25 volunteers whose ABI had not been measured and 51 who either were or had been taking drugs for hyperlipidemia, or were uncertain about whether they were or had been taking such drugs, 473 participants (219 women, 254 men) were enrolled in our study. Serum CyPA levels in 195 randomly selected participants were detected using enzyme-linked immunosorbent assays (ELISA).

### Ankle brachial index

We made the diagnosis of PAOD by measuring the ABI. Each participant was first asked to lie down and rest for 5 to 10 minutes. Then, the brachial pressures and ankle pressures of the dorsalis pedis arteries and posterior tibial arteries were measured using a hand-held Doppler device and a blood pressure cuff. First, the blood pressure cuffs were placed about 1 inch above the antecubital fossa on both arms and about 2 inches above the medial malleolus on both ankles. Second, the Doppler probes were used to detect the arterial pulse signals, and then the cuffs were inflated until the arterial Doppler signals disappeared. Finally, the systolic pressure for each vessel was measured by recording the points where the Doppler signals reappeared during deflating. To calculate the ABI of each side, the higher value of the two ankle pressures in each leg was divided by the higher value of the two brachial pressures. The participant’s ABI was defined as the lower value^22^.

### PAOD diagnosis

PAOD was diagnosed when a participant’s ABI was less than 0.91, based on the diagnostic criteria of the American College of Cardiology Foundation/American Heart Association (ACCF/AHA) in 2011. A value between 0.91 and 1.40 is normal. A value greater than 1.40 indicates that the leg artery is stiff and noncompressible^23^.

### Renal function assessed and chronic kidney disease diagnosed

The renal function was evaluated using the estimated glomerular filtration rate (eGFR), which was calculated by testing serum creatinine (SCr) levels and using the Cockcroft-Gault (CG) formula (23):

CG = [(140-age) × weight × 0.85 if female]/(72 × SCr), adjusted for BSA by 1.73 m^2^/BSA

CKD was diagnosed when a participant’s eGFR value was less than 60 ml/min per 1.73 m^2^ based on the National Kidney Foundation’s Kidney Disease Outcome Quality Initiative (NKF-KDOQI)^23^.

### Statistical analysis

SPSS for Windows (SPSS Inc., Chicago, IL) was used for the following analysis. Significance was set at p < 0.05. Based on the ABI values, participants were divided into three groups: PAOD, normal arteries, and noncompressible arteries. In our current study, we compared only between subjects with PAOD (ABI ≤ 0.90) and with normal ABI (0.91–1.40). Basic characteristics, medical histories, and biochemistry parameters were compared. Those continuous variables with normal distribution were compared using a Student’s *t* test; otherwise, a non-parametric Mann-Whitney U test was used. Categorical variables were compared using χ^2^ tests. The independent effects of BMI, HbA1c, eGFR, and pulse pressure were further evaluated using logistic regression and expressed as odds ratios (ORs) and 95% confidence intervals (CI). CyPA levels were compared using a non-parametric Mann-Whitney U test, and bivariate analysis was used to explore the association between CyPA and ABI and between CyPA and PAOD risk factors.

## Results

We finally enrolled 473 participants (mean age: 76.1 ± 0.3 years, range: 65–102 years) and divided them into three groups based on their ABI values: 68 participants (14.4%) were diagnosed with PAOD (≤ 0.90), 392 (82.9%) were normal (0.90–1.40), and 13 (2.7%) had noncompressible arteries (≥ 1.41). The prevalence of PAOD was 14.4%. In our current study, we compared subjects with PAOD (ABI ≤ 0.90) and with normal ABI (0.90–1.40).

### Associations between PAOD and traditional cardiovascular risk factors

In the PAOD group, the HbA1c level was significantly higher and BMI was significantly (both p < 0.05) lower than in the normal group. There were no significant differences in the gender, the prevalence of hypertension or smoking, or the lipid profile between these two groups (Table 1).

### Associations between PAOD and non-traditional cardiovascular risk factors

The prevalence of CKD was 22.2% in this elderly population. The renal function (eGFR) was significantly impaired amount the PAOD group (ABI 0.91–1.4 vs. ABI < 0.91: 90.2 ± 37.0 vs. 72.0 ± 32.2 mL/min/1.73 m^2^, p = 0.001). In addition, pulse pressure was significantly higher in the PAOD group than in the normal group (56.5 ± 15.0 vs. 62.3 ± 16.4 mmHg, p = 0.008) (Table 1).

### Multiple risk factors analysis

**Figure 1:**
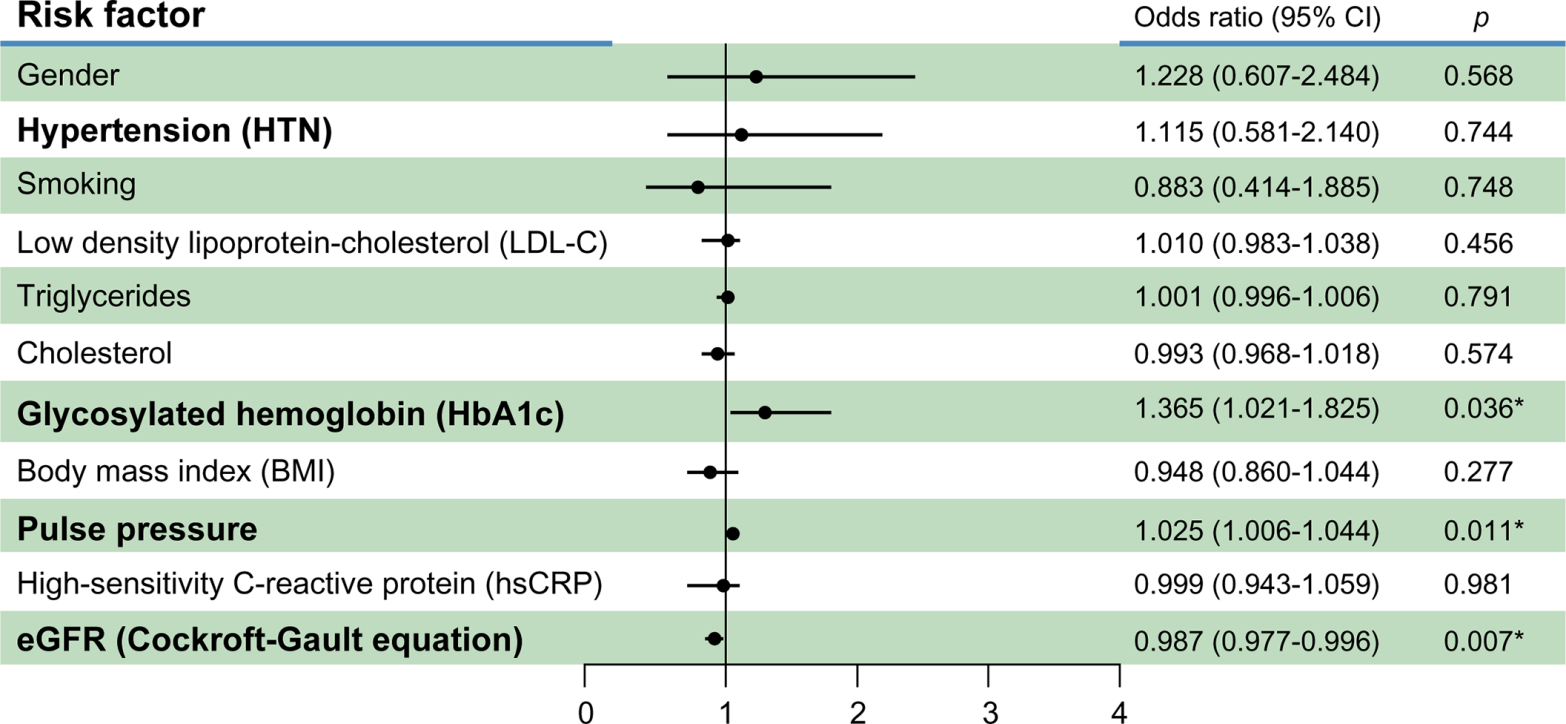
Multiple logistic regression analysis showed that glycosylated hemoglobin (HbA1c), pulse pressure, and estimated glomular filtration rate (eGFR) were associated with peripheral arterial occlusive disease (PAOD) (ABI ≤ 0.90).

All PAOD risk factors were added to the logistic regression analysis as covariates. HbA1c (OR: 1.365, 95% CI: 1.021–1.825, p = 0.036), pulse pressure (OR: 1.025, 95% CI: 1.006–1.044, p = 0.011), and eGFR (OR: 0.987, 95% CI: 0.977–0.996, p = 0.007) were independently associated with PAOD (Figure 1).

### The associations between CyPA, PAOD and risk factors

**Figure 2:**
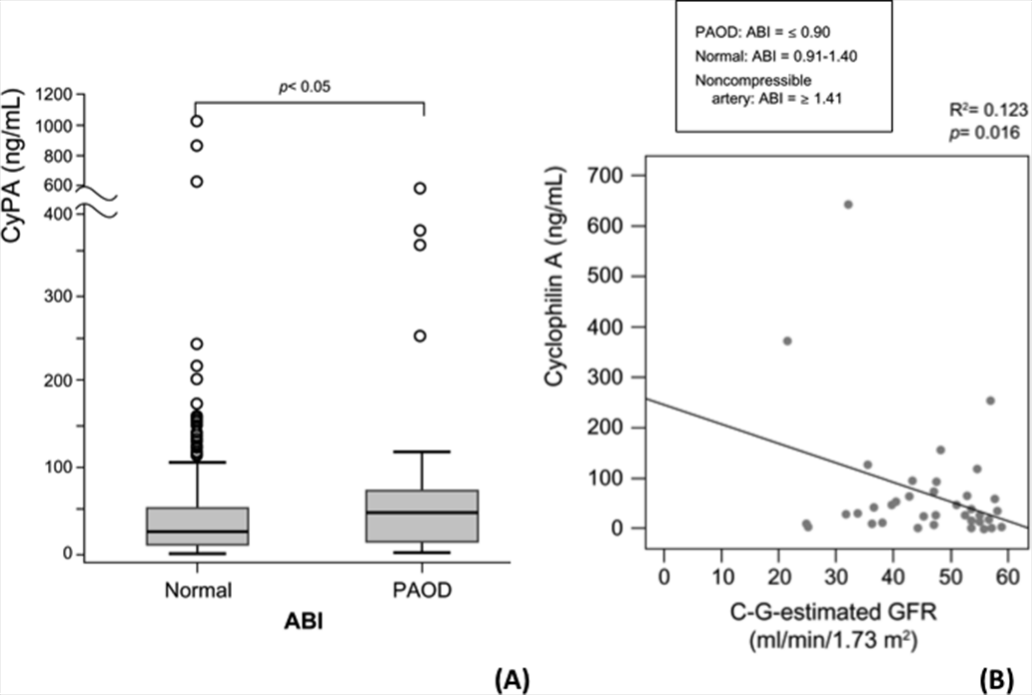
Serum level of cyclophilin A (CyPA) was higher in the PAOD (peripheral arterial occlusive disease) group (ankle brachial index [ABI] ≤ 0.90) and was associated with renal function only in the chronic kidney disease (CKD) subgroup. (A) Participants with PAOD had a significantly higher median of serum CyPA; (B) Serum CyPA level and estimated glomular filtration rate (eGFR) were inversely correlated in participants with CKD.

Serum CyPA levels were measured in 195 randomly selected with a sex-and-age matched 1:1 participants. Those with PAOD (n = 68) had higher median serum CyPA levels than did those with normal ABI values (47.3 ± 0.4 vs. 25.5 ± 0.2 ng/mL, p < 0.05) (PAOD vs. normal subjects; serum CyPA mean ± SD value: 83.6 ± 44.7 vs. 56.1 ± 50.3 ng/mL) (Figure 2A).

**Figure 3:**
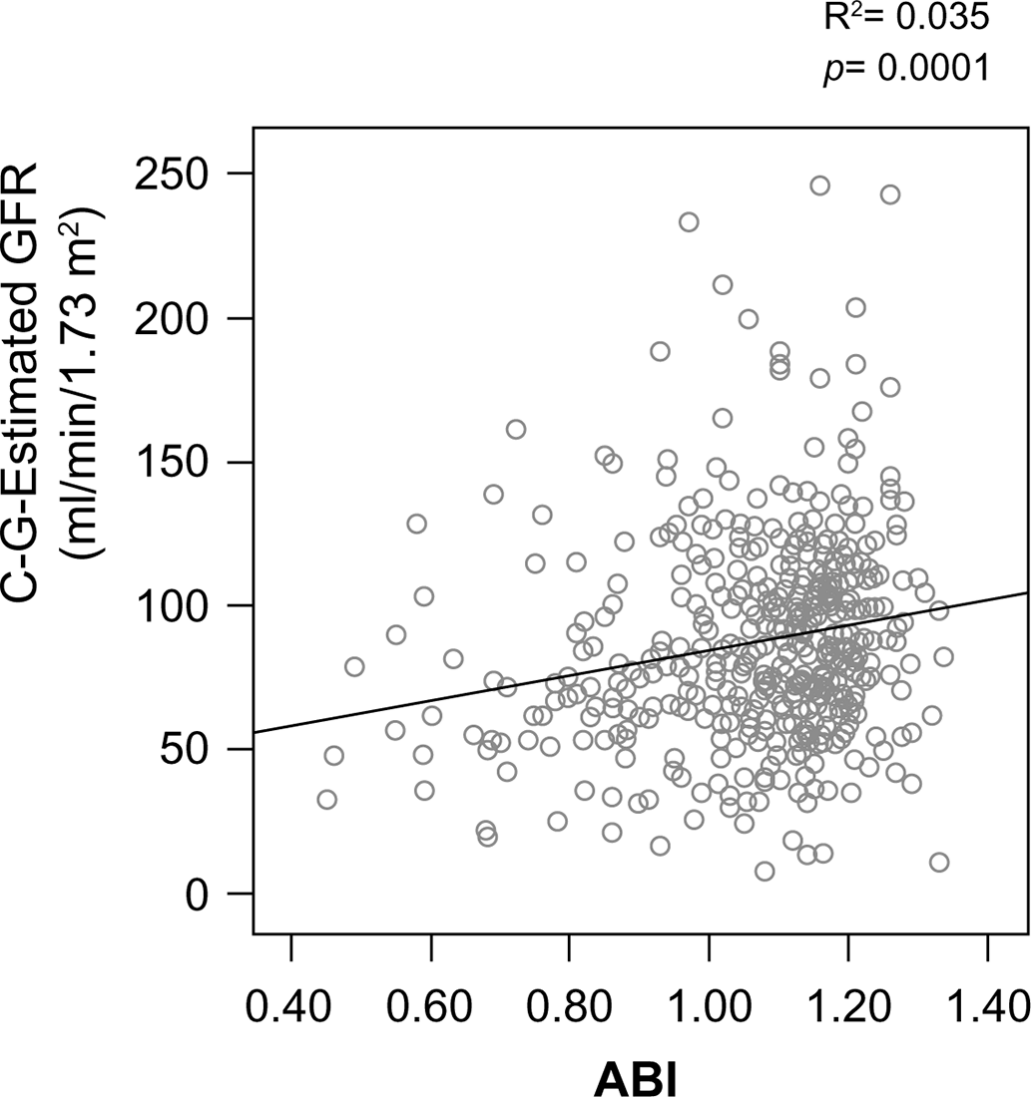
Estimated glomular filtration rate (eGFR) was positively correlated with ankle brachial index (ABI) value.

The association between eGFR and serum CyPA levels was not significant for all the participants. Interestingly, once we subdivided them into 2 groups based on whether they had CKD (eGFR < 60 mL/min/1.73 m^2^, n = 49), there was an inverse correlation between eGFR and serum CyPA level (p = 0.016) (Figure 2B). In addition, the lower eGFR was, the more severe the PAOD was (p = 0.0001) (Figure 3). Interestingly, this was not true for participants with a normal eGFR.

## Discussion

We found that the prevalences of both PAOD and CKD were high in the elderly residents of Tianliao District. Significant correlation was found between advanced PAOD and a higher HbA1c, impaired renal function and increased pulse pressure. Moreover, there was an interesting interplay between PAOD, CKD, and CyPA.

### Traditional risk factors for PAOD

The prevalence of PAOD in our elderly participants was 14.4%, lower than that in the German Epidemiological Trial on Ankle Brachial Index (getABI) study^24^ (21%), but it was similar to what was reported by the Lipid Research Clinics (LRC) Prevalence Study^25^ (11.3%) and the Cardiovascular Health Study^7^ (13%).

HbA1c, pulse pressure, and eGFR were independently associated with PAOD in our participants. HbA1c is a tool for evaluating the long-term blood glucose level and diagnosing diabetes. Both type I and types II diabetes are risk factors for atherosclerosis because they lead to prolonged exposure to hyperglycemia^26^. We found that participants with PAOD had a higher HbA1c, which was an independent risk for PAOD, and that there was a similar result in the Atherosclerosis Risk in Communities study^27^. Patients with diabetes and comorbid PAOD had a higher HbA1c in the ARIC study. A higher HbA1c indicated poor blood sugar control, and can lead to atherosclerosis. HbA1c was a PAOD risk factor contributed by multiple mechanisms, including the increased production of advanced glycosylation end products, increased oxidative stress burden, and the activation of protein kinase C^26^.

Widening pulse pressure, which is frequently considered an indication of large artery stiffness caused by loss of elastin, is common in the elderly^27^. In addition to being an independent risk for PAOD, pulse pressure was inversely correlated with ABI in the current study. These results support reports^28^,^29^,^30^ that widening pulse pressure is associated with PAOD as both a cause and a consequence of atherosclerosis^31^. Other studies^32^,^33^ have shown that endothelial dysfunction is a crucial step in the progression of atherosclerosis, and that widening pulse pressure can damage conduit artery endothelial function.

We found an association between reduced eGFR and PAOD. In addition, eGFR was positively correlated with ABI, which is evidence of the association between PAOD and impaired renal function^7^. CKD was an important disease in our participants. The prevalence of CKD in Taiwan ranged from 9.83% to 11.93% between 2003 and 2008, and it was even higher in this elderly population (22.2%)^5^,^34^. Over the past few decades, CKD has been increasingly associated with the development of cardiovascular disease, especially atherosclerosis^23^. The mechanism is still not clear; however, that CKD and atherosclerosis share the risk factors of HTN, DM, hypercholesterolemia, obesity, and oxidative stress may be an explanation^35^.

### Serum CyPA, PAOD, and CKD

Oxidative stress is involved in the pathogenesis of atherosclerosis^36^. The present study found evidence to support its hypothesis that the level of serum CyPA, which can be considered a product of oxidative stress and a pro-atherogenic molecule, is associated with HbA1c, pulse pressure, and eGFR. In addition, serum CyPA level increases and renal function declines once CKD has developed. In contrast, however, serum CyPA level was inversely correlated with eGFR in 49 participants with CKD. Although additional research is required to explore and confirm the role of CyPA in the pathophysiology of CKD, these results indicated that as a pro-atherogenic molecule.

Previous studies^37^,^38^,^39^ showed that CypA could be actively released into the extracellular milieu by a number of cell types, including macrophages^37^, vascular endothelial cells^38^, and vascular smooth muscle cells^39^. *In vivo*, extracellular CypA concentrations were elevated in rodent models of asthma^40^ and acute lung injury^41^. In blood vessels, concentrations were also increased during vascular remodeling^42^, and mice lacking the gene for *CypA*, commonly referred to as *Ppia2/2* mice, will be protected from aortic aneurysm formation^43^. To sum up, the CyPA could be stimulated by several cell types and can be a causative molecular for vascular pathology^44^. Whether extracellular CyPA was excreted by kidney remained unclear. In addition, epidemiologic and clinical evidence supports that subjects with advanced renal dysfunction usually had higher oxidative stress, inflammatory status. We assumed that CyPA might affect vasculature via either by the increased secretion from the kidney, or by an enhanced serum level from several cell types stimulation, indirectly due to the CKD environment. In our current study, however, we did not measure the level of urine CyPA, further studies were required to explore the correlation of serum CyPA and urine CyPA.

### Limitations

This study has some limitations. First, although several interesting correlations were found, determining a cause-effect relationship is not possible in a cross-sectional study. Second, the number of participants whose serum CyPA level was tested was small. Third, our participants were volunteers; therefore, only ambulatory residents were surveyed. Consequently, the prevalence of PAOD might be underestimated. Fourth, we did not perform and complete OGTT or other tests for the whole population to confirm the possible “occult” or pre-DM subjects. Also, we did not record the detail information on diabetic kidney disease from participants during this community-based survey. This might cause a covariant bias because diabetes is an important contributor for whole CKD population. In addition, we did not measure the urine CyPA to correlate the association between serum CyPA, urine CyPA and their roles pathophysiologically.

## Conclusion

We found that the prevalence of PAOD in one Taiwan elderly population was similar to that reported in other studies, and that HbA1c, pulse pressure, and eGFR were significantly associated with PAOD. Serum CyPA level might be a link between PAOD and impaired renal function in CKD. Future studies of CyPA in CKD were needed to confirm the correlation between CyPA and impaired renal function in larger population and also determine the cause and effect of CyPA and CKD vascular disease.

## Author Contributions

Conceived and designed the experiments: P.Y.L. and C.H.W. Collected and analyzed the data: M.C.L., Y.W.L., P.T.L., C.S.C., J.R.Y., X.T.S., L.W.H. and Y.L.T. Biostatistics analysis: S.H.L. Prepared the Tables and Figure: M.C.L. and P.Y.L., Wrote the paper: M.C.L. and P.Y.L. All authors reviewed the manuscript.

## Figures and Tables

**Table 1:** Basic characteristics of the geriatric cohort, subdivided by ankle brachial index values

	Ankle Brachial Index	
Characteristics and variables	≤ 0.90	0.91–1.40
	PAOD (n = 68)	Normal (n = 392)
Gender (female)	34 (50%)	184 (46.9%)
Diabetes mellitus (DM)	18 (26.5%)	70 (17.9%)
Hypertension (HTN)	57 (83.8%)	335 (85.5%)
Current or former cigarette smoker	18 (26.5%)	103 (26.3%)
Total cholesterol (mg/dL)	205.5 ± 36.8	202.7 ± 37.1
Triglycerides (mg/dL)	116.0 ± 0.3	106.0 ± 0.1
LDL cholesterol (mg/dL)	130.7 ± 31.4	127.0 ± 32.2
HDL cholesterol (mg/dL)	50.5 ± 14.9	51.0 ± 12.8
Hemoglobin A1c (HbA1c) (%)*	5.9 ± 0.3	5.8 ± 0.1
Body mass index (BMI)* (kg/m^2^)	23.3 ± 3.4	24.5 ± 3.8
Systolic blood pressure (SBP) (mmHg)	136.4 ± 21.9	133.8±20.0
Diastolic blood pressure (DBP)* (mmHg)	74.1±11.1	77.4±11.6
Pulse pressure (mmHg)*	62.3 ± 16.4	56.5 ± 15.0
hsCRP (mg/L)	1.1 ± 0.3	1.2 ± 0.1
eGFR (mL/min/1.73 m^2^)*	72.0 ± 32.2	90.2 ± 37.0

PAOD = peripheral occlusive artery disease; eGFR = estimated glomerular filtration rate; HDL = high-density lipoprotein; hsCRP = high-sensitivity C-reactive protein; LDL = low-density lipoprotein.

*p < 0.05 compared with PAOD (≤ 0.90) and Normal (0.91–1.40) groups.

Categorical variables are presented as n (%). Continuous variables with normal distribution are presented as mean ± standard deviation (SD). Continuous variables with non-normal distribution (triglycerides, HbA1c, and hsCRP) are presented as median ± standard error (SE).
